# From Jane Doe to Sofia: DNA Extraction Protocol from Bones and Teeth without Liquid Nitrogen for Identifying Skeletal Remains

**DOI:** 10.3390/ijms25105114

**Published:** 2024-05-08

**Authors:** Emanuela Stan, Camelia-Oana Muresan, Raluca Dumache, Veronica Ciocan, Stefania Ungureanu, Alexandra Mihailescu, Ecaterina Daescu, Corina Duda-Seiman, Gheorghita Menghiu, Delia Hutanu, Alexandra Enache

**Affiliations:** 1 Department of Neuroscience, Discipline of Forensic Medicine, Bioethics, Deontology and Medical Law, “Victor Babes” University of Medicine and Pharmacy, 300041 Timisoara, Romania; emanuela.stan@umft.ro (E.S.); raluca.dumache@umft.ro (R.D.); veronica.luta@umft.ro (V.C.); stefania.ungureanu@umft.ro (S.U.); enache.alexandra@umft.ro (A.E.); 2 Institute of Legal Medicine, 300610 Timisoara, Romania; alexandra.mihailescu@umft.ro (A.M.); daescu.ecaterina@umft.ro (E.D.); 3 Ethics and Human Identification Research Center, Department of Neurosciences, “Victor Babes” University of Medicine and Pharmacy, 300041 Timisoara, Romania; 4 Department of Microscopic Morphology Genetics, Center of Genomic Medicine, “Victor Babes” University of Medicine and Pharmacy, 300041 Timisoara, Romania; 5 Department I of Anatomy and Embryology, “Victor Babes” University of Medicine and Pharmacy, 300041 Timisoara, Romania; 6 Doctoral School Medicine-Pharmacy, “Victor Babeș” University of Medicine and Pharmacy, 300041 Timișoara, Romania; 7 Faculty of Chemistry-Biology-Geography, West University of Timisoara, 300115 Timișoara, Romania; gheorghita.menghiu@e-uvt.ro (G.M.); delia.hutanu@e-uvt.ro (D.H.)

**Keywords:** forensic anthropology, forensic genetics, skeletal DNA extraction, autosomal STR typing, DNA profiles, human identification

## Abstract

DNA analysis plays a crucial role in forensic investigations, helping in criminal cases, missing persons inquiries, and archaeological research. This study focuses on the DNA concentration in different skeletal elements to improve human identification efforts. Ten cases of unidentified skeletal remains brought to the Institute of Forensic Medicine in Timisoara, Romania, underwent DNA analysis between 2019 and 2023. The results showed that teeth are the best source for DNA extraction as they contain the highest concentration of genetic material, at 3.68 ng/µL, compared to the petrous temporal bone (0.936 ng/µL) and femur bone (0.633 ng/µL). These findings highlight the significance of teeth in forensic contexts due to their abundant genetic material. Combining anthropological examination with DNA analysis enhances the understanding and precision of identifying human skeletal remains, thus advancing forensic science. Selecting specific skeletal elements, such as the cochlea or teeth, emerges as crucial for reliable genetic analyses, emphasizing the importance of careful consideration in forensic identification procedures. Our study concludes that automated DNA extraction protocols without liquid nitrogen represent a significant advancement in DNA extraction technology, providing a faster, more efficient, and less labor-intensive method for extracting high-quality DNA from damaged bone and tooth samples.

## 1. Introduction

Every skeleton whispers the secret of the life and death of its owner. Starting from a bone, the forensic anthropologist restores the identity of “Jane Doe” to the families so those suffering for their loved ones can find relief and mourn them.

One of the most crucial aspects of forensic genetics is the identification of human remains, particularly those of missing individuals. DNA analysis, a powerful tool in various forensic scenarios, plays a pivotal role in this process. From aiding in criminal investigations by identifying perpetrators to resolving cases of missing persons, DNA-based human identification is invaluable. Its applications extend to archaeological research, mass fatality incidents, and parentage questioning, underscoring its wide-ranging importance [1,2].

Since 1985, when Jeffreys et al.’s research revealed how to identify individuals through DNA fingerprint analysis and used DNA polymorphism detection for the first time in a forensic investigation, DNA profiling has developed into a powerful tool in identification investigations [3]. DNA analysis has seen a rapid evolution due to the necessity for identification methods that are more sensitive, precise, and require less inferior-quality material. PCR-based methods have enabled the targeting of different genetic markers, ranging from single-nucleotide polymorphisms to varying numbers of tandem repeats [4]. In forensic genetics, short tandem repeats (STRs), most often genotyped to discriminate between individuals, are the most commonly used technique of personal genetic identification. STR profiling is the gold standard in human forensic identification [5]. According to Budowle and van Daal [6], the forensically significant STR loci usually have five to twenty common alleles. High polymorphic information content values and a strong capacity for individual discrimination distinguish these STRs [7]. In addition to its usefulness in crime scene investigation, STR profiling is an essential tool in human identification that can be utilized in maternity and paternity testing, disaster victim identification, and kinship testing [5].

Environmental factors contributing to DNA degradation play a significant role in preserving DNA in skeletal remains. In addition to the time that has passed since an organism died, the environment in which it was located after death significantly impacts DNA preservation. The main environmental elements that influence degradation include the soil’s geological and chemical characteristics, radiation exposure, pH, oxygen and moisture access, the presence of microorganisms, and temperature [8,9]. As a result, samples may be limited, deteriorate, or contain impurities interfering with the ability to type DNA samples.

Skeletons are frequently incomplete and fragmented; therefore, sampling many skeletal components for genetic studies is not always feasible. Although teeth are regarded as the best source of DNA, they may not always be accessible for testing. They have the advantage of having an exterior structure that reduces microbial activity and safeguards the DNA inside [10,11]. Bones are occasionally the only source of accessible DNA due to their structure, which retains DNA effectively and for an extended period. Some researchers think that nuclear DNA acts as a matrix during fibrillogenesis and that DNA’s interaction with Type I collagen may help keep DNA stable [12]. Other research has shown that DNA can attach to hydroxyapatite [13]. Additionally, during the mineralization process, it is also feasible that DNA fragments could become trapped and encased within hydroxyapatite crystallites [14].

According to current recommendations for sampling forensic structures, the most appropriate bones for isolating and analyzing DNA from skeletons are compact ones, particularly long bones (the femur and tibia) and teeth. Conversely, flat and spongy bones, like the skull, vertebrae, and ribs, are less suitable [10,15]. The best skeletal component for sampling prehistoric skeletal remains, according to Pinhasi et al., is the petrous section of the temporal bone [16]. Pilli et al. report similar findings, demonstrating that DNA is better preserved in the petrous portion of the temporal bone than in the femur and teeth. They conclude that this results from the high bone density in the temporal bone’s petrous region, which increases resistance and reduces bacterial DNA damage [17].

Genetic procedures used for the forensic identification of people rely on comparing the material under investigation with reference samples. Reference samples can be collected more often from the victim’s items or the victim’s known biological relatives [18]. Among the reference samples from living biological relatives, we encountered the most frequent samples from relatives in a direct line, either parents or children (son, daughter), or, in the absence of these, even more distant blood relatives on the paternal or maternal line.

Through molecular technology, the identities of thousands of bone remains were identified, enabling families to bury their loved ones respectfully [19].

This research aimed to analyze several forensic cases in which identifying unknown remains was the central objective of the investigations. The primary purpose was to investigate DNA concentration depending on the type of bone being examined and to illustrate the advantages of DNA human identification from various human bone remains. Another goal of this study was to integrate both the anthropological examination and the DNA analysis to assess the identification of human skeletal remains.

## 2. Results

In two cases, anthropological analyses were conducted as the first step in the identification process before genetic identification.

### 2.1. Anthropological Examination of Jane Doe 2

**Figure 1:**
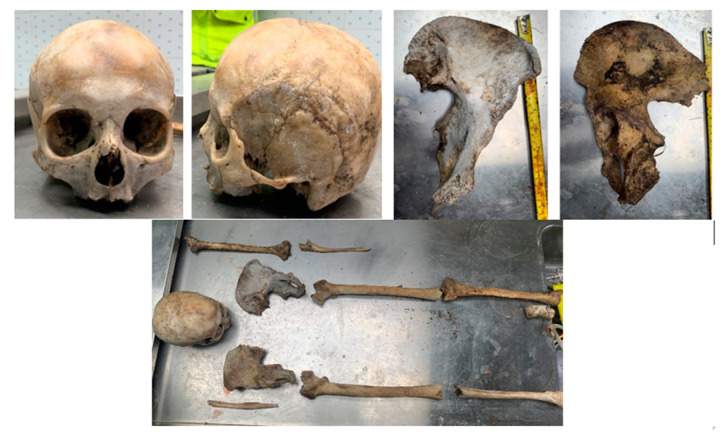
Case 2: Skeletal remains of Jane Doe 2—anthropological examination.

Upon examination of the skeletal remains attributed to Jane Doe 2, a preliminary anthropological analysis was undertaken to establish key characteristics aiding in her identification. The examination encompassed 16 bone fragments of non-human origin, a human skull lacking a mandible, and 11 bone fragments sourced from the postcranial skeleton (Figure 1). Given certain pelvic and cranial characteristics typical of female morphology, it was assumed that Jane Doe 2 was an adult and most likely female over 65 years old. Following a thorough examination, it was concluded that the person’s death was most likely the result of natural causes rather than external trauma because there were no indications of injury or critical trauma, and the body could have belonged to someone who passed away approximately six months ago after being exposed to external environmental conditions. In summary, although the anthropological investigation provided helpful insights into the demographic profile and circumstances surrounding Jane Doe’s death, the fragmented condition of the skeletal remains made it difficult to reach solid conclusions. These initial discoveries established the foundation for future genetic identification endeavors focused on determining the person’s identity.

### 2.2. Anthropological Analysis of Jane Doe 3

**Figure 2:**
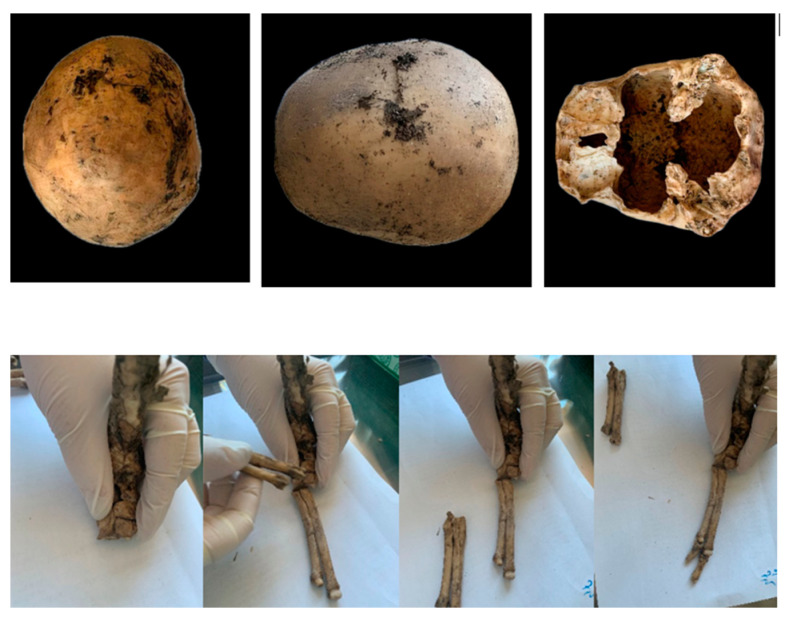
Skeletal remains of Jane Doe 3—anthropological examination. Human fragmented skull and non-human bones.

Morphological features characteristic of sexual dimorphism were scrutinized to ascertain the sex of the individual. Although it was challenging to determine Jane Doe 3’s sex with certainty due to the skeletal elements’ fragmented nature, the observed traits suggested that she was most likely an adult female. In addition to the human skeletal remains, non-human bone fragments were identified within the assemblage. Through comparative analysis, these fragments were attributed to an animal from the canid family, potentially indicative of a canine origin, such as a dog (Figure 2). Examination of the analyzed skull fragment revealed no discernible evidence of vital trauma or injury. The skeletal remains displayed features indicative of a postmortem interval of around four to five months, corresponding to the timeframe from March to April 2019. Based on the finding of the remains in late July 2019, it can be deduced that the person most likely died during that time and was then exposed to external environmental conditions.

Subsequent to the anthropological examination, a fragment of the petrous pyramid of the skull was collected for the extraction of genetic material.

The quantity of human DNA detected with real-time PCR in the skeletal remains after quantification using the Power Quant^®^ System (Promega Corp., Madison, WI, USA) is shown in Table 1.

Comparative analyses were conducted between the genetic profiles obtained from the bones and the reference sample. The probabilistic and statistical interpretation of the results was carried out using the GenoProof 3-Parentage Examination software version 3.0.4 (Qualitype, Dresden, Germany) according to the Paternity Testing Commission of the International Society of Forensic Genetics recommendations.

**Case 1:** Genetic analysis of biological samples extracted from the femur diaphysis fragment of Jane Doe 1 yielded a complete genetic profile indicative of a male individual. Comparative analysis between this genetic profile and that of the presumed biological father (S1) confirmed paternity with a high probability (PP = 99.9999498%) and a paternity index (PI = 41.021 × 10^3^). These results conclusively established the biological relationship between Jane Doe 1 and the presumed father.

**Case 2:** The genetic analysis of biological samples extracted from the femur diaphysis fragment of Jane Doe 2 yielded a complete genetic profile consistent with a female individual. Upon comparative analysis with the genetic profile of the alleged son (S2), a high probability of maternity (PM = 99.9965%) and a maternity index (MI = 2.918) were observed. These significant findings conclusively establish the biological relationship between Jane Doe 2 and the alleged son, thereby confirming the identity of the skeletal remains as those of Jane Doe 2, underscoring the importance of our analysis in confirming identities.

**Case 3:** DNA identification was performed using a fragment of the dense portion of the petrous bone, situated outside the otic capsule, from Jane Doe 3. Analysis of biological samples extracted from this fragment yielded a unique genetic profile complete with all analyzed DNA markers indicative of a female individual. The comparative study of the two genetic profiles indicates that the probability of kinship of Jane Doe 3, investigated as the presumed mother of the S3, is confirmed on all DNA markers analyzed, with the probability of maternity (PM) = 99.999254159%.

**Figure 3:**
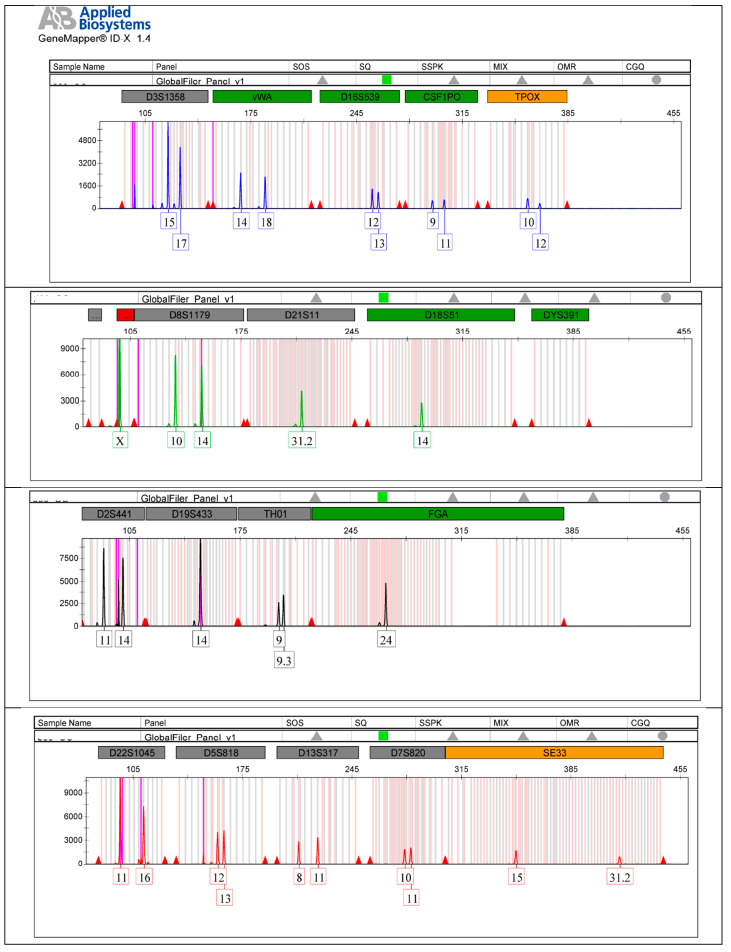

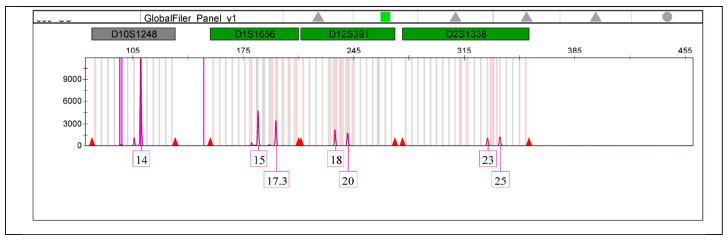
The autosomal STR electropherogram of Jane Doe 3.

**Figure 4:**
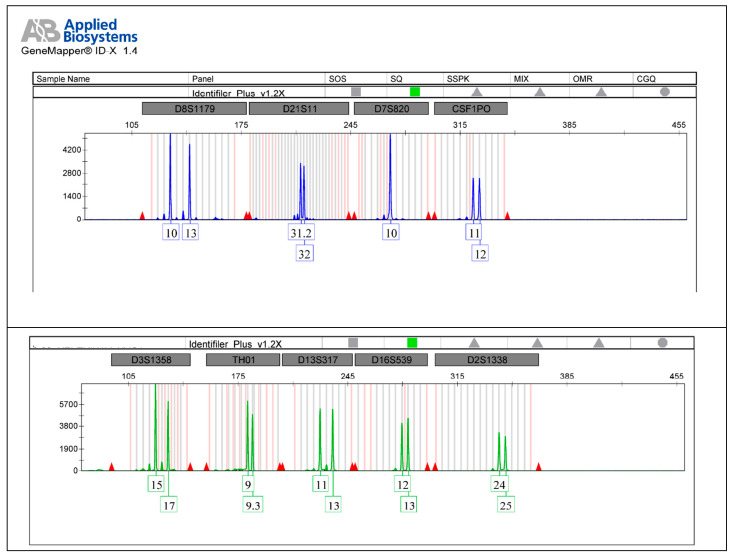

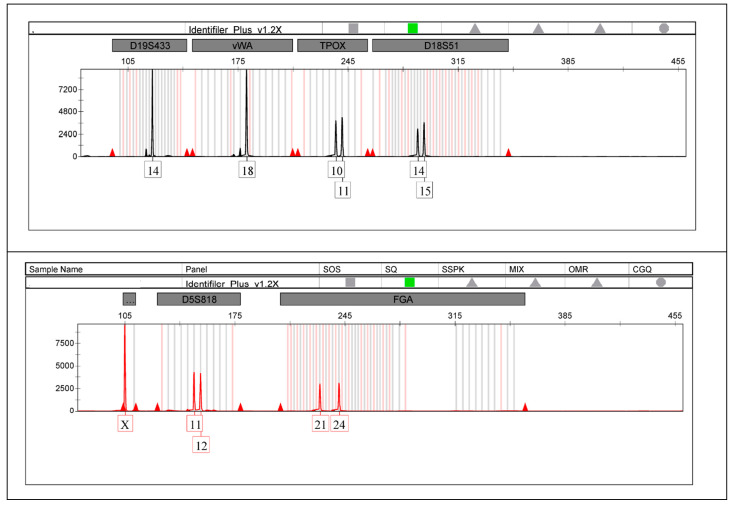
The autosomal STR electropherogram of alleged daughter of Jane Doe 3 from the reference sample.

The autosomal STR electropherograms of Jane Doe 3 (Figure 3) and her alleged daughter (Figure 4) are analyzed using the Gene Mapper ID-X software version 1.4 (Applied Biosystems, Waltham, MA, USA).

**Case 4:** DNA analysis was conducted using biological samples extracted from the dense bone of the otic capsule (cochlea) of John Doe 4, resulting in the acquisition of a unique genetic profile complete on all analyzed markers indicative of a male individual. A comparative analysis was performed between the genetic profile obtained from the cochlea and the alleged son (S4) reference sample. The results revealed a high paternity probability (PP = 99.99936%) and a paternity index (PI = 346.12 × 10^2^), confirming the biological relationship between John Doe 4 and the alleged son.

**Case 5:** Genetic analysis of biological samples obtained from the canine tooth belonging to John Doe 5 revealed a unique genetic profile, complete on all analyzed autosomal DNA markers, indicative of a male individual. Comparative analysis between this genetic profile and that of the alleged son (S5) confirmed paternity with a high probability (PP = 99.999989%) and a paternity index (PI = 1.36 × 10^2^). Additionally, genetic analysis of the same biological sample from the canine tooth of John Doe 5 yielded a unique genetic profile complete on all analyzed Y-STR markers, further supporting the male lineage. A comparative study of the Y-STR haplotypes between John Doe 5 and the alleged son (S5) revealed an identical match, providing unequivocal evidence of a father–son biological relationship between the two individuals (Table 2).

**Case 6:** DNA analysis was carried out using biological samples extracted from John Doe 6’s rib, which resulted in the acquisition of a unique genetic profile that was complete on all analyzed markers and indicative of a male individual. A comparative analysis was then conducted between the genetic profile obtained from the rib and that of S6, the alleged brother’s reference sample. The analysis showed a biological relationship between the two individuals with a probability of a biological relationship of 92.911152537%.

**Case 7:** A genetic profile was obtained from a femur fragment taken from John Doe 7’s biological samples. The genetic profile belongs to a male person and is complete on all analyzed markers. After comparing it with the genetic profile of the alleged son, a high probability of paternity (PM = 99.9999999%) and a paternity index (PI = 819,011 × 10^3^) were observed. These findings conclusively establish the biological relationship between John Doe 7 and the alleged son, confirming the identity of the skeletal remains as those of John Doe 7.

**Case 8:** According to the results obtained from the genetic analysis, it has been demonstrated that there is a biological father–daughter relationship between the presumptive daughter D8 and the genetic profile of John Doe 8 from the clavicle fragment. The paternity (PP) probability is 99.99999%, and the index of paternity (PI) is 18.003990 × 10^−7^. Additionally, the genetic analysis comparison between John Doe 8 and the presumed son S8 shows a biological father–son relationship with a paternity (PP) probability of 99.99999% and an index of paternity (PI) of 22,005,270 × 10^−5^.

**Case 9** involved genetic analysis of biological samples extracted from a femur diaphysis fragment of John Doe 9. The results indicate that the individual was a male. A comparative analysis was conducted between the genetic profile of John Doe 9 and the presumed son (S9). The analysis confirmed paternity with a high probability (PP = 99.99993458%) and a paternity index (PI = 235.56 × 10^3^). These conclusive results establish a biological father–son relationship between John Doe 9 and the presumed son S9.

**Case 10:** DNA analysis was conducted using biological samples extracted from the teeth of John Doe 10 (taken by the railway from the place of the accident). This resulted in the acquisition of a unique genetic profile complete on all analyzed markers indicative of a male individual. The obtained genetic profile corresponds to the reference profile of the deceased (taken during the forensic autopsy).

## 3. Discussion

When conducting genetic identification of skeletal remains, it is crucial to consider the relationship between the types of bones and the DNA concentration obtained during analysis. Based on the amount of DNA that can be extracted, various studies have classified the recommended types of human bones for forensic genetic identification, such as the tooth, talus, tarsal bones, petrous temporal bone, vertebra, femur, and tibial metatarsal [20].

Our knowledge of the composition and decay process of bones and teeth is crucial in selecting appropriate samples for DNA analysis in skeletal remains. Dental pulp, which has good blood flow and nerve innervation, is an important source of DNA. Teeth that have larger pulps and several roots are considered the best sources of DNA since they have more pulp cells and dental cementum than teeth with only one root. Molars are considered to be the best teeth for DNA extraction since they have larger pulps and several roots, providing more pulp cells and dental cementum than teeth with only one root [9]. DNA extracted from teeth usually has higher quality than DNA extracted from bones. Additionally, complete genotypes are most frequently obtained from femurs and teeth due to the superior quality of DNA extracted from these bones and teeth [19].

Studies have shown that the petrous part of the temporal bone may offer even more potential as a substrate for DNA extraction than teeth due to its dense composition and specific structure [21]. The percentage of DNA obtained from petrous bone is four–sixteen times higher than that obtained from teeth and significantly superior to that obtained from other skeletal elements, such as ribs, metacarpals, or metatarsal bones [22].

The selection of the bone type best suitable for DNA extraction in forensic practice stirred numerous debates. In a tree case study, Corrêa et al. [23] highlight that femur samples provide equal or inferior results compared to cuneiform bones or distal foot phalanges, recovering 84.9% versus 99.2%, and 96.8%, accordingly, of true alleles.

Bones represent a relatively challenging sample for forensic analysis due to scarce and poorly preserved DNA. Most classical techniques are quite laborious and require pulverizing the bone fragments, thus requiring an easier and more feasible approach. In another study, Corrêa et al. [24] performed a comparative analysis of DNA extraction from bone powder versus a protocol using large fragment demineralization. Their study was performed on 30 fragments of femoral or tibial diaphysis obtained from cadavers in advanced stages of decomposition. The analysis showed that large fragment demineralization can be useful as a complementary tool in STR analysis, as in one case, it was capable of yielding more alleles compared to the protocol using bone powder. Additionally, no statistically significant differences were observed in peak heights between the protocol involving bone pulverization versus fragment demineralization.

Research findings on samples from Holocene archaeological contexts in Eurasia, dated between 10,000 and 1800 years before the present, confirm the hypothesis that the densest part of the petrous bone, composing the otic capsule, yielded the best results. The yields from the dense bone of the otic capsule (inner ear), encompassing the cochlea, vestibule, and three semicircular canals, are up to 65 times higher than those from the dense bone portion of the petrous bone outside the otic capsule. Additionally, they are up to 177 times higher than those from the bone at the top of the petrous pyramid, which is primarily trabecular. Total endogenous DNA concentrations are up to 126-fold and 109-fold higher for these comparisons. Therefore, even though dense petrous portions can provide significant endogenous yields, bone samples collected straight from the otic capsule yield exceptionally high endogenous DNA percentages [16].

The findings of our study provide valuable insights into the efficacy of different skeletal elements for DNA extraction in forensic identification. Our results support the hypothesis that the dense bone of the otic capsule, particularly the cochlea, yields superior DNA concentrations compared to other skeletal elements. Specifically, we observed a significantly higher DNA concentration of 3.30 ng/µL from the cochlea, highlighting its potential as an optimal source for DNA extraction in forensic investigations.

On the other hand, genetic tests on samples from the dense bone part of the petrous outside the otic capsule showed a lower DNA concentration of 0.561 ng/µL. This disparity underscores the importance of selecting the most suitable skeletal elements for DNA extraction to maximize the likelihood of obtaining high-quality genetic profiles for identification purposes.

Our study found that teeth contain the highest DNA concentration among the skeletal elements we analyzed. DNA concentration measured from teeth was 3.68 ng/µL, which is higher than the concentrations obtained from petrous temporal bone and femur bone samples, which yielded DNA concentrations of 0.936 ng/µL and 0.633 ng/µL, respectively. These results indicate that teeth may be the best source for DNA extraction in forensic contexts, as they provide high yields of genetic material for reliable identification efforts.

Furthermore, our study demonstrates the importance of using multiple genetic markers, such as autosomal DNA markers and lineage markers like Y-STRs, to improve identification accuracy in forensic cases. Specifically, in the case of John Doe 5, an unidentified individual found in an advanced stage of decomposition, we were able to use both autosomal and Y-STR markers to establish a biological relationship, thanks to the availability of a reference sample from an alleged son. Based on previous research, we decided to incorporate both autosomal and Y-STR markers, which has shown that combining these marker types is effective in human identification, especially when reference samples from male family members are available [25]. Dumache et al. [26] have also emphasized the usefulness of using autosomal STR markers and Y-STR markers to enhance the accuracy and reliability of identification efforts involving missing or unknown persons.

The utilization of highly effective DNA extraction techniques is paramount, particularly when dealing with damaged DNA materials. In forensic contexts, where DNA yields may be anticipated to be low due to the state of the samples, it is crucial to employ extraction methods that maximize the quality and quantity of the extracted DNA [26].

The use of liquid nitrogen in forensic laboratories during the grinding process of bone demineralization has been a common practice. However, with advancements in DNA extraction technology, new kits have been introduced that are specifically designed for DNA extraction from severely deteriorated bones. One example of such a kit is the one introduced by Promega Company in 2019. This kit has significantly improved DNA extraction and PCR amplification processes, enabling forensic laboratories to obtain accurate results with minimal damage to bone samples and a reduced DNA extraction time of only three hours [27,28]. In this study, DNA extraction from bones and teeth samples was conducted without using liquid nitrogen, in accordance with the protocol used in the Genetic Laboratory of Forensic Institute of Medicine Timisoara, Romania [29]. Our results show that it is feasible and effective to use an automated DNA extraction protocol without liquid nitrogen. This approach not only simplifies the extraction process but also accelerates the extraction of high-quality bone powder from bones while preserving DNA samples. By implementing automated DNA extraction protocols without the need for liquid nitrogen, forensic laboratories can streamline their workflows and expedite the DNA extraction process. This is particularly beneficial in cases where time is of the essence, such as in rapid response scenarios or high-volume casework environments. Additionally, the use of such protocols minimizes the risk of sample contamination and reduces the potential for operator error, ultimately improving the overall efficiency and reliability of forensic DNA analysis.

We acknowledge that DNA research has inherent challenges and that the risk of contamination is a valid concern. Bones, as a source of DNA, can be particularly problematic due to factors such as degradation and potential contamination. Therefore, DNA extraction from bone is meticulous and precise, especially in identification, where accuracy is of the utmost importance. Even the smallest amounts of foreign DNA can compromise the accuracy of results and potentially lead to false conclusions in forensic investigations.

Smajlovic-Skenderagi et al. [30] have studied the issue of human remains contamination by bacterial DNA and its impact on genetic testing using STR markers in human remains identification. Bone and teeth samples from skeletal remains retrieved from mass graves from Bosnia and Herzegovina and Kosovo accounted for most of the samples analyzed during this study. Other samples came from victims of natural disasters in Thailand and the Maldives and missing person identification cases from Iraq, Cyprus, Brazil, Kuwait, and Chile. Their findings suggest that bacterial peaks are easily distinguishable from true peaks in most cases because they do not fall within allelic ladder bins or have a particular morphology. Challenges are represented by cases in which the bacterial peaks match the size of a true allele, and the sample is homozygous for the chosen locus. Specialists in forensic genetics should consider these aspects in order to obtain and interpret the results accurately.

It is inevitable that microbial DNA is co-extracted and occasionally co-amplified with targeted human DNA sequences due to the great diversity and prevalence of microbes. It is commonly known that human DNA isolated from skeletal samples copurifies with microbial DNA, permeating the bone matrix and remaining for extended periods of time. Progress has been achieved in removing microbial DNA from bone samples. Techniques like bleach pre-treatment and sodium phosphate washes have shown a favorable reduction in the copurification of bacterial DNA, even though some endogenous DNA is lost in the process [31,32].

To address the contamination issue, we have implemented stringent protocols to minimize DNA contamination throughout our experiments. We conduct DNA extraction and genotyping in a certified forensic genetics laboratory that has obtained an International Society for Forensic Genetics (ISFG) Certificate of Excellence for DNA testing at international standards. This ensures that forensic scientists can maintain the integrity and reliability of DNA evidence in criminal investigations. In addition, the forensic genetic laboratory has specialized tools and facilities for DNA extraction and analysis, including a DNA cleanroom. Our controlled environment has strict measures to minimize airborne particles, microbes, and other contaminants and includes physical separation of pre- and post-PCR workspaces. Our personnel follow established guidelines for DNA research because they must ensure the integrity of the DNA extraction process and avoid contamination. To combat this threat, they diligently follow strict protocols, wear protective clothing, use sterile instruments, and adhere to meticulous decontamination procedures for equipment and reagents. They work in designated clean areas like DNA clean rooms. This thorough approach, the use of special equipment and facilities, and adherence to strict contamination prevention measures are crucial for upholding the accuracy and validity of forensic findings in legal proceedings.

Although using DNA for identification may not be groundbreaking and is not a new concept, this article provides an analysis of DNA typing from skeletal remains specifically tailored to address the unique challenges and requirements of forensic investigations in our country. The study offers insights into selecting appropriate bones for DNA extraction and comparative DNA yields from different skeletal elements, ultimately enhancing forensic investigations involving skeletal remains and delivering justice in our country.

## 4. Materials and Methods

### 4.1. Case Selection and Sample Collection

Our study focused on analyzing modern DNA from bone fragments of ten unknown skeletal remains (Table 3), which belonged to individuals who had passed away between a few days and two years ago prior to the DNA analysis. The remains were brought to the Institute of Forensic Medicine in Timisoara, Romania, between 2019 and 2023 for DNA identification purposes. The cases were selected based on the availability of well-preserved skeletal samples and the requirement for DNA analysis to establish positive identification. Standard forensic procedures were followed to collect skeletal samples, including femur (long bones), cranium, and teeth, to prevent contamination and preserve the DNA.

A preliminary anthropological investigation was conducted to examine the bones’ anthropological characteristics, pathological abnormalities, and trauma signs and to determine variables such as sex, stature, and age where possible.

### 4.2. DNA Extraction Protocol

The bone fragments undergo a thorough preparation process for DNA analysis, involving specialized equipment and facilities, including a DNA cleanroom dedicated to bone samples submitted for forensic examination and analysis. This is undertaken to maintain the purity of the DNA samples and adhere to strict contamination prevention measures. DNA extraction and genotyping are carried out in a certified forensic genetics laboratory, using specialized equipment and facilities to prevent contamination. This area is monitored for DNA contamination regularly.

### 4.3. Bone Preparation

The bone remnants that were gathered underwent a washing process utilizing a coarse portion of a dish sponge that had been sterilized and exposed to UV radiation. Subsequently, the bones were washed three times in sterile bi-distilled water (Merck Millipore^®^, Merck KgaA, Darmstadt, Germany), employing a gentle detergent (a small amount of detergent, equivalent to a 5% volume of water). The bone fragments that had been washed were allowed to dry overnight. Subsequently, the bone powder was acquired through the process of grinding the bones into small fragments at a frequency of 30 Hz for 1–2 min, utilizing the Bead Beater Mill Mix 20 manufactured by Tehtnica Domel in Slovenia (Domel Holding, d.d., Železniki, Slovenia).

### 4.4. DNA Extraction

After drilling to obtain a bone powder, samples must be decalcified to liberate DNA from the mineral bone matrix. The buffer for demineralization was prepared using the Bone DNA Extraction Kit, Custom (Promega Corp., Madison, WI, USA), following the FSC DNA IQ Maxwell protocol. According to the protocol, each sample must include 100 milligrams of bone powder and use two bone lysis cocktails to release DNA. The first bone lysis cocktail contains 400 µL of demineralization buffer, 40 µL of proteinase K, and 10 µL of 1-thyoglicerol, while the second cocktail is composed of 990 µL lysis buffer added to 10 µL 1-thyoglicerol. The sample with the first bone lysis cocktail was left to incubate in the Eppendorf ThermoMixer C (Eppendorf, Hamburg, Germany) for 2.5 h, with intermittent shaking at 1000 rpm. After that, the sample was centrifugated at 13.300 rpm for 5 min, after which the supernatant was transferred into another sterile 1.5 mL Eppendorf tube. Reference DNA samples from the deceased’s family members were extracted using the Maxwell^®^FSC DNA IQTM Casework (Promega Corp., Madison, WI, USA) on the automated Maxwell^®^ 48 RSC instrument (Promega Corp., Madison, WI, USA). After extraction, all samples containing eluted DNA were stored at T = 4 °C prior to quantification.

### 4.5. DNA Quantification

In order to determine the quantity of nuclear DNA, the DNA extracts from bone samples were quantified by real-time PCR using the Power QuantTM System Kit (Promega Corp., Madison, WI, USA). A mix solution with a total volume of 18 µL was prepared for each sample, consisting of 10 µL of Power Quant 2X Master Mix, 7 µL of Amplification Grade Water, and 1 µL of Power Quant 20X Primer Mix. The 7500 Real-Time PCR Equipment (Applied Biosystems, Waltham, MA, USA) with HID Real-Time PCR Analysis Software, version 2.0.6 (Applied Biosystems, Waltham, MA, USA), was used to perform the reactions following the manufacturer’s instructions. 

### 4.6. Amplification of the DNA Samples

Target sequences were amplified starting from extracted DNA, using a thermocycler ProFlex PCR System (Applied Biosystems, Waltham, MA, USA) and the AmpFLSTR Identifier Plus PCR Amplification Kit (Applied Biosystems, Waltham, MA, USA) or Global FilerTM PCR Amplification Kit (Thermo Fisher Scientific, Waltham, MA, USA). In case 5, along with the AmpFLSTR Identifier Plus PCR Amplification Kit (Applied Biosystems, Waltham, MA, USA), the AmpFLSTR Y filer (Applied Biosystems, Waltham, MA, USA) was used.

Simultaneously with the forensic samples, we amplified the positive control and negative PCR controls.

The quality of DNA extracts obtained from the bone powder was further evaluated by analyzing the STR profiles generated from the STR amplification procedure. Both the extraction and PCR negative controls showed no signs of contamination. We successfully amplified DNA from all the bones analyzed, and complete genetic profiles were acquired.

### 4.7. Visualization and Identification of Reaction Products

Further, visualization and identification of reaction products were performed by capillary electrophoresis and automatic LASER fluorescent detection using the ABI 3500 genetic analyzer (Applied Biosystems, Waltham, MA, USA). Data analysis was conducted using Gene Mapper ID-X Software version 1.4 (Applied Biosystems, Waltham, MA, USA). DNA profiles obtained from the bones and teeth of our unidentified subjects were analyzed and compared to those obtained from living relatives. DNA genotypes from living relatives were obtained by analyzing the DNA isolated from saliva.

Data interpretation was carried out with the help of the GenoProof 3-Parentage Examination software, version 3.0.4 (Qualitype, Dresden, Germany) according to the recommendations of the Paternity Testing Commission of the International Society of Forensic Genetics.

## 5. Conclusions

Our research underscores the crucial role of carefully selecting skeletal elements for DNA extraction in forensic identification procedures. The use of specific skeletal elements, such as the cochlea or teeth, can significantly impact the reliability and success of genetic analyses. This, in turn, can facilitate the accurate identification of individuals in forensic investigations. Our study concludes that automated DNA extraction protocols without liquid nitrogen are a practical and effective method for forensic laboratories. This approach represents a significant advancement in DNA extraction technology, providing a faster, more efficient, and less labor-intensive method for extracting high-quality DNA from damaged bone and tooth samples. Ultimately, this innovation enhances the capabilities of forensic DNA analysis.

## Figures and Tables

**Table 1:** Sample type and DNA concentrations (ng/μL).

Case No.		Type of Sample	DNA Quantification Method/Kit Name	DNA Concentrations (ng/μL)
Case 1	alleged father	femur diaphysis fragment	PowerQuant (Promega Corp., Madison, WI, USA)	0.936
	presumptive daughter D1	saliva		10.73
Case 2	Jane Doe 2	femur diaphysis fragment	PowerQuant (Promega Corp., Madison, WI, USA)	0.633
	presumptive son S2	saliva		12.35
Case 3	Jane Doe 3	skull—petrous bone	PowerQuant (Promega Corp., Madison, WI, USA)	0.561
	presumptive daughter D3	saliva		21.94
Case 4	John Doe 4	skull fragment—cochlea	PowerQuant (Promega Corp., Madison, WI, USA)	3.30
	presumptive daughter D4	saliva		27.35
Case 5	John Doe 5	canine tooth	PowerQuant (Promega Corp., Madison, WI, USA)	3.68
	presumptive son S5	saliva		5.93
Case 6	John Doe 6	rib fragment	PowerQuant (Promega Corp., Madison, WI, USA)	1.678
	presumptive brother B6	saliva		34.926
Case 7	John Doe 7	femur diaphysis fragment	PowerQuant (Promega Corp., Madison, WI, USA)	3.67
	presumptive son S7	saliva		12.84
Case 8	John Doe 8	clavicle fragment	PowerQuant (Promega Corp., Madison, WI, USA)	5.78
	presumptive son S8 presumtive daughter D8	saliva saliva		24.78 21.56
Case 9	John Doe 9	femur diaphysis fragment	PowerQuant (Promega Corp., Madison, WI, USA)	1.895
	presumptive son S9	saliva		13.26
Case 10	John Doe 10 (found at the accident place)	tooth	PowerQuant (Promega Corp., Madison, WI, USA)	2.72
	John Doe 10 (autopsy)	biological soft tissue		1.06

**Table 2:** Case 5: Genetic profiles (haplotypes) determined on Y-STR markers of the presumptive son and Jane Doe 5.

Markers Y-STR	Genetic DNA Profile of the Presumptive Son	Genetic DNA Profile Obtained from the Canine Tooth
DYS456	16	16
DYS389I	12	12
DYS390	21	21
DYS389II	29	29
DYS458	17	17
DYS19	15	15
DYS385	13;16	13;16
DYS393	14	14
DYS391	11	11
DYS439	13	13
DYS635	22	22
DYS392	11	11
Y GATA	11	11
DYS437	16	16
DYS438	10	10
DYS448	23	23

**Table 3:** Information concerning unidentified skeletal remains cases.

Case No.	Location/Found	Material Harvested for DNA Extraction	Reference Sample
1	cemetery—exhumation	a diaphysis fragment of the left femur	buccal swab (presumed daughter)
2	some bone fragments and clothing items on an empty lot next to the residence where she lives	skull	buccal swab (presumed son)
3	human remains had been spotted in the woods in a shopping cart	skull—petrous bone	buccal swab (presumed daughter)
4	advanced putrefaction stage	skull—cochlea	buccal swab (presumed daughter)
5	advanced putrefaction stage (abandoned storage)	canine tooth	buccal swab (presumed son)
6	carbonized body in house fire	rib	buccal swab (presumed brother)
7	carbonized	femur	buccal swab (presumed son)
8	carbonized (house fire)	clavicle	buccal swab (presumed son and daughter)
9	carbonized	femur	buccal swab (presumed son)
10	train accident (high fragmentation)	tooth	buccal swab (from the deceased during autopsy)

Note: Reference samples were collected from living relatives or individuals with a potential familial relationship to the unidentified remains.

## Data Availability

The data presented in this study are available on request from the corresponding author. The data are not publicly available due to confidentiality reasons.
